# Curcumin and Its Derivatives as Potential Therapeutic Agents in Prostate, Colon and Breast Cancers

**DOI:** 10.3390/molecules24234386

**Published:** 2019-11-30

**Authors:** Zintle Mbese, Vuyolwethu Khwaza, Blessing Atim Aderibigbe

**Affiliations:** Department of Chemistry, University of Fort Hare, Alice Campus, Alice 5700, Eastern Cape, South Africa; zintlembese@gmail.com (Z.M.); vuyolwethukhwaza@gmail.com (V.K.)

**Keywords:** curcumin, anticancer activity, derivatives of curcumin, drug resistance, breast cancer, prostate cancer, colon cancer

## Abstract

Cancer is a life-threatening disease and is the second leading cause of death around the world. The increasing threats of drug-resistant cancers indicate that there is an urgent need for the improvement or development of more effective anticancer agents. Curcumin, a phenolic compound originally derived from turmeric plant (*Curcuma longa* L. (Zingiberaceae family)) widely known as a spice and a coloring agent for food have been reported to possess notable anticancer activity by inhibiting the proliferation and metastasis, and enhancing cell cycle arrest or apoptosis in various cancer cells. In spite of all these benefits, the therapeutic application of curcumin in clinical medicine and its bioavailability are still limited due to its poor absorption and rapid metabolism. Structural modification of curcumin through the synthesis of curcumin-based derivatives is a potential approach to overcome the above limitations. Curcumin derivatives can overcome the disadvantages of curcumin while enhancing the overall efficacy and hindering drug resistance. This article reports a review of published curcumin derivatives and their enhanced anticancer activities.

## 1. Introduction

**Figure 1:**
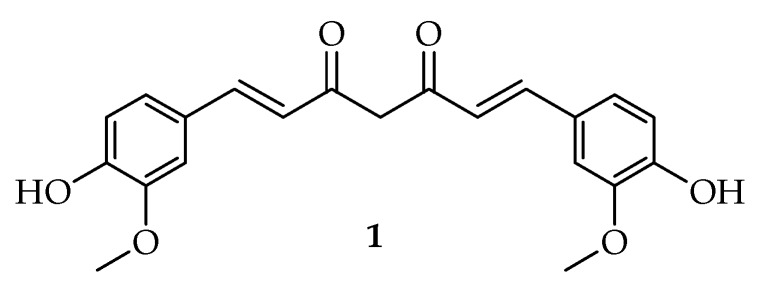
Structure of curcumin.

Curcumin (Figure 1) is one of the most important components of the curcuminoids family [1]. It is also called as diferuloylmethane, which can be isolated from the rhizome of *Curcuma longa* L. [2]. It was first discovered in 1815, though its chemical structure was identified in 1973 by Roughley and Whiting with a melting point ranging from 176 °C to 177 °C [3,4]. Curcumin is known to be the most effective, safe, non-toxic, and main bioactive components available in turmeric, and it also exhibits a range of biological actions [5]. The main problem with curcumin is its poor bioavailability and low absorption [3]. Hence, various researchers are concentrating on improving its bioavailability, therapeutic efficacy, and pharmacological properties for the treatment of human diseases through many methods, including the development of novel curcumin derivatives [6,7]. Structural modification of curcumin results in compounds with multiple biological activities suitable for the treatment of various diseases, such as cardiovascular diseases, diabetes, neurodegenerative diseases, etc. Therapeutically, curcumin and its derivatives are widely used as potential anticancer, anti-inflammatory, antimicrobial and anti-oxidants agents. The efforts to synthesize novel curcumin derivatives with enhanced biological activities have been reported [1,8,9]. Clinical trials have shown that the biological activity of drugs, such as curcumin, can be achieved by improving the activity of the drug [10]. Researchers have found that curcumin possesses anticancer properties because of its effect on many biological pathways involved in mutagenesis, oncogene expression, tumorigenesis, cell cycle regulation, apoptosis, and metastasis [11,12]. Therefore, to improve the limitations and increase the anticancer activities of curcumin, extensive efforts have been continuously devoted to the syntheses of new curcumin derivatives [13,14,15]. Curcumin derivatives exhibited several anticancer activities in cancer cell lines, such as prostate [16], breast [17], and colon cancer cells [9,18]. This review is focused on curcumin and its derivatives with enhanced anticancer activities on breast, prostate, and colon cancer.

## 2. Anticancer Activity of Curcumin and Its Derivatives

Cancer is a chronic disease characterized by the development of abnormal cells that spreads and destroys normal body tissue [19]. It is one of the most leading life-threatening diseases globally. According to United States statistics in 2018, new cases of cancer were about 1.73 million with more than 609,000 deaths [20]. The IARC (International Agency for Research on Cancer) estimated that new cancer cases are expected to increase. In 2008, 12.7 million new cancer cases were reported globally, 5.6 million cases were from economically developed countries, and about 7.1 million cases were from economically developing countries. The total estimate for cancer deaths in 2008 was 7.6 million (about 21,000 cancer deaths a day), 2.8 million in economically developed countries and 4.8 million in the economically developing countries [21]. Due to the million cases of cancer deaths mentioned above, there is an urgent need to develop new and potent anticancer agents. Many researchers have highlighted the effectiveness of natural products in the development of anticancer drugs [19]. Natural compounds, such as resveratrol, Epigallocatechin-3-gallate (EGCG), and curcumin, have been recently shown to be effective in chemoprevention [22,23,24]. However, their use is limited by poor bioavailability. They have been reported to exhibit additive effect when combined with anticancer drugs resulting in synergistic effects. Curcumin has no noticeable toxicity, and the existing data suggest that curcumin combination with chemotherapeutic agents is a superior approach for the treatment of colon cancer [25]. Curcumin displayed similar results of efficacy in vitro as oxaliplatin, an anticancer drug at therapeutically achievable concentrations in both p53 mutant and wild type colorectal carcinoma cell lines [26]. Furthermore, curcumin has been reported to be extremely safe, even at relatively high doses in various animal models and clinical studies [27,28]. Chen et al. investigated the anticancer activity of curcumin in vitro and in vivo and the results revealed the potent inhibitory effect of curcumin on carcinogenesis at three stages: angiogenesis, tumor promotion, and tumor growth [25]. The National Institute of Cancer (NIC) nominated curcumin as an anticancer agent [21]. In 1987, it was the first time the anticancer activity of curcumin was reported using human subjects. A clinical trial was performed on 62 different patients with external cancerous lesions [29].

Curcumin has a wide range of biological activities, including anti-inflammatory, antimicrobial, antioxidant, and anticancer, antidiabetic activities [30]. Hence, curcumin is known as a promising drug in treatment of human diseases, such as infectious diseases, cancer, neurodegenerative diseases, and diabetes. However, the use of curcumin is limited due to the following factors [31]:

- Low aqueous solubility;
- Instability in aqueous condition;
- Poor bioavailability;
- Poor cellular uptake.

The aforementioned limitations hinder the clinical application of curcumin [31]. To overcome these limitations and improve the overall potent anticancer properties of curcumin, several approaches, such as the synthetic route, for curcumin derivatives must be considered to improve its selective toxicity towards specific cancer cells [32]. A number of studies have focused on modifying the structure of curcumin with the aim of enhancing curcumin derivatives with improved bioavailability and enhanced specific biological activities [23,26,27]. Curcumin derivatives inhibit tumor proliferation, metastasis, growth, invasion, and angiogenesis and cause damage in apoptosis-resistant cells [15].

### 2.1. Advantages of Using Curcumin Derivatives

**Figure 2:**
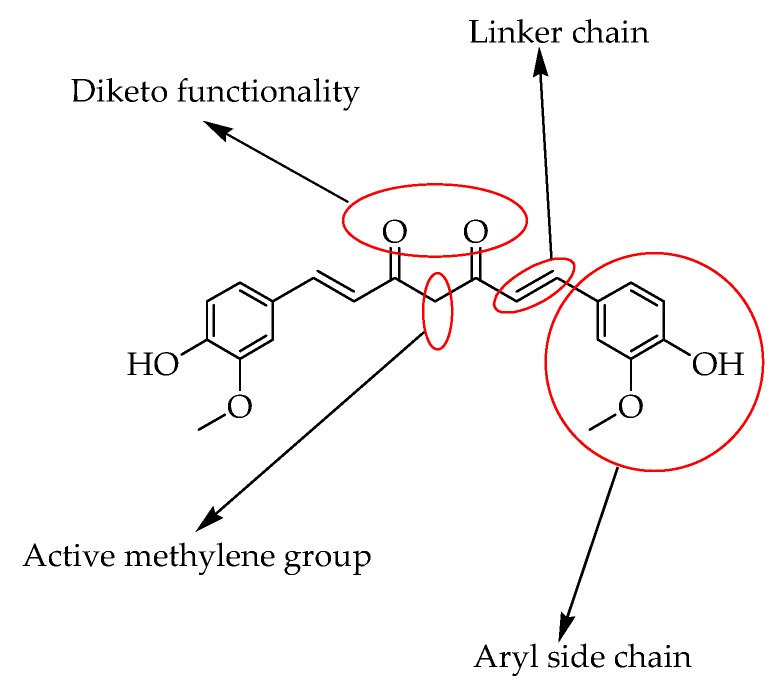
Curcumin structure indicating the major reactive sites.

Curcumin and its derivatives have received huge attention because of their biological actions such as anti-inflammatory, antioxidant and antitumor activities. The mentioned agents are ascribed to the important elements for the structure of curcumin. Structurally, curcumin is a symmetrical molecule comprising of four chemical entities, aryl side chains which are linked together by a linker in the presence of a diketo functional group, two double bonds, and an active methylene moiety (Figure 2). Each of these sites tend to be a potential site for suitable modifications to improve the efficacy of curcumin.

**Figure 3:**
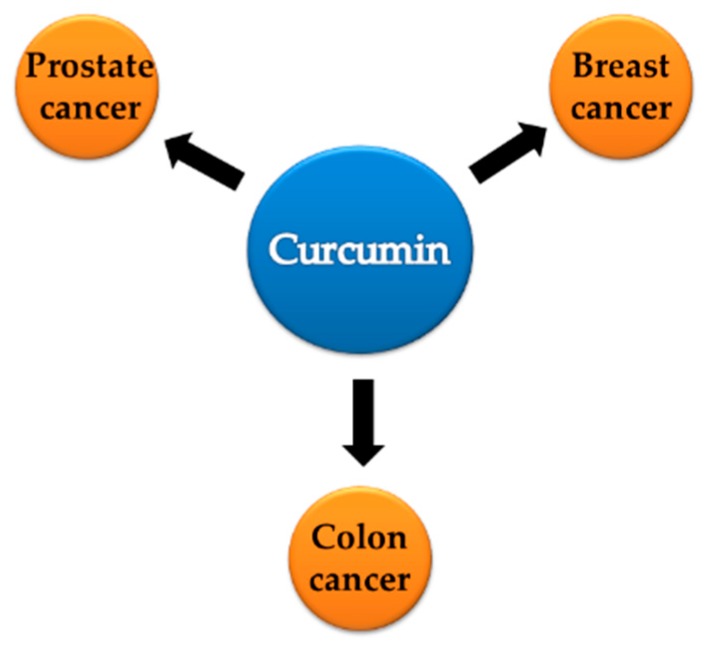
Three selected different types of cancers inhibited by curcumin.

Modifying the chemical structure of curcumin does not only improve the pharmacological activity of a drug molecule and affect the receptor binding but also improve its physiochemical and pharmacokinetic properties [17,28,29,30]. Some other derivatives have shown improved antitumor and anti-inflammatory actions when compared to curcumin because of the high level of methoxylation, the unsaturation of the diketone moiety, and a low level of hydrogenation. When the curcumin compound is compared to its analogs, it shows a powerful antioxidant activity for many hydrogenated curcumin analogs [31,32]. Moreover, curcumin derivatives possess higher cytotoxicity against numerous tumor cell lines when compared to normal cells (Figure 3) [33].

### 2.2. Resistance to the Currently Used Medicine

The problem with the currently used anticancer drugs in chemotherapy is toxicity to normal cells. To reduce the toxic side effect, it is essential to reduce the doses. Hence, the improvement of anticancer drug with reduced toxicity and low side effects has turned into a principal objective in numerous immune–pharmacology studies [1]. The currently used anticancer drugs suffer from drug resistance. Many studies have shown that cancer stem cells are the key to cancer drug resistance. Cancer stem cells possess an effective efflux of anticancer drugs through the ATP (Adenosine Triphosphate)-binding cassette (ABC) transporters which are the main cause of development of drug resistance in cancer. ABC transporters are the members of the superfamily which transports various substrates through the membrane (extracellular and intracellular) and serve as the potential player in innate and acquired multi-drug resistance (MDR) of many cells including cancer stem cells. The ABC transporters (ABCG2), act as a resistance marker in both cancer stem cells and cancer cells and help in the determination of prognosis of malignancies and also drug bioavailability [34,35]. The synthesis of hybrid compounds as potential therapeutic agents is more effective when compared to using a single bioactive agent. Since drug resistance is a major problem with the currently used anticancer drugs, many studies are focused on the design of potent anticancer agents that can overcome the problem of drug resistance which is common with most anticancer drugs [36].

### 2.3. Mode of Action of Curcumin Derivatives

Curcumin demonstrates unique antitumor activity, such as inhibiting proliferation, cell survival pathway, inducing apoptosis, death receptor pathway, and protein kinase pathway, to inhibit the tumor growth and invasion of cancers by suppressing different types of cellular signaling pathways [20]. Additionally, curcumin is effective at different phases of cancer development. It blocks the transformation of cancer cells, it prevents normal cells before they can be able to form tumors (tumor initiation), metastasis, angiogenesis, and invasion. Curcumin anticancer activity in vivo and in vitro revealed its capability to suppress carcinogenesis and also prevent the proliferation of many types of tumor cells [37]. The different mechanisms of action of curcumin and its molecular targets have been summarized in Table 1.

## 3. Three Different Types of Cancers

### 3.1. Breast Cancer

In females, breast cancer is the second most common leading cause of deaths among cancers worldwide [54]. Despite chemotherapy, lumpectomy, endocrine therapy, and radiation therapy, the rate of deaths in breast cancer is still high and increasing. Hence, it is essential to design effective therapeutic agents [14]. Studies demonstrated that the role of cancer stem cells is very significant, more especially, in breast cancer because these cells are able to control cancer formation, resistance, and progression to therapy [8]. Some natural products with low toxicity and various biological properties have been used as an alternative for the treatment of cancers such as breast cancer. Since breast cancer is known as the common cancer in females worldwide, the statistics of cancers account for approximately 25% with a higher prevalence in developed countries [15]. Most researchers also demonstrated that curcumin possesses anticarcinogenic and antiproliferative activities in a broad spectrum of tumor tissues and animals. Additionally, current studies reveal that curcumin when used in combination with other anticancer drugs can efficiently induce apoptosis [15,55,56].

#### 3.1.1. Curcumin Derivatives as Breast Cancer Inhibitors

**Scheme 1:**
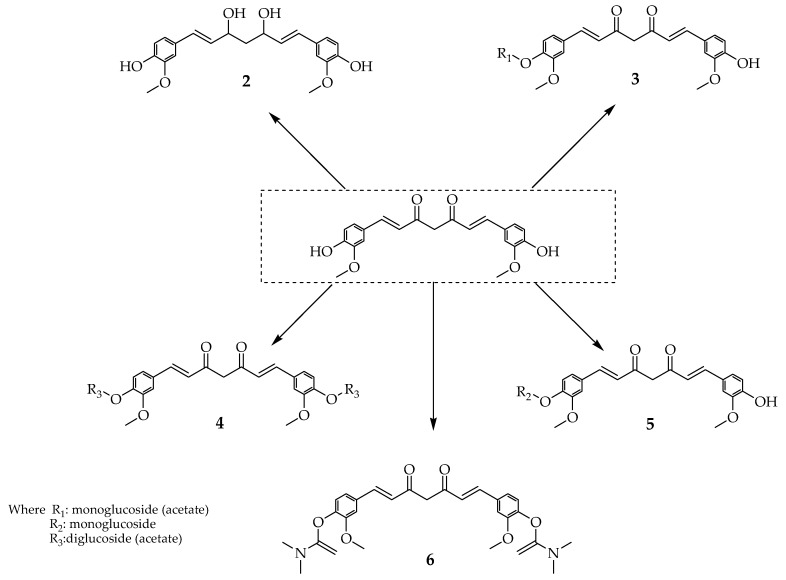
Compounds **1**–**6**.

**Scheme 2:**
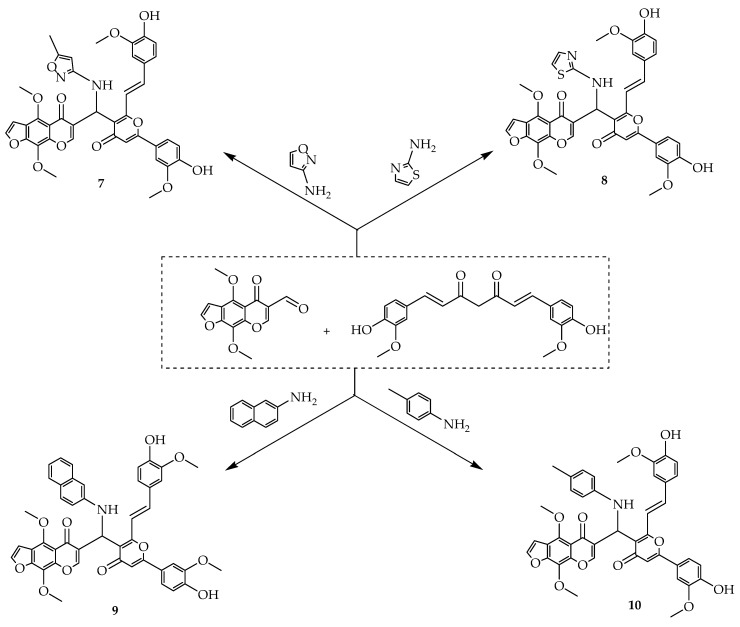
Compounds 7–10.

Tripathi and Misra synthesized a series of new curcumin derivatives with potential synergistic anticancer activity which inhibits the growth of breast cancer stem cells by hindering the P-glycoprotein (P-gp) mediated efflux mechanism (Scheme 1). Glucoside of curcumin derivatives has shown a higher binding affinity towards P-gp when compared to other curcumin derivatives resulting in the reduction of the growth of breast cancer stem cells [8]. The current anticancer drugs that are being used for chemotherapy are toxic to normal cells; hence, they cause toxicity towards immune cells. Therefore, it is essential to reduce doses to the smallest amount and also to reduce the side effects of these drugs [1]. It is important to develop novel anticancer drugs with low or no side effects on the immune system. Scheme 2 (Compound **7**–**10**) shows heterocyclic curcumin derivatives that were synthesized by Borik et al. Their cytotoxic effect against breast carcinoma (MCF-7) cell lines was evaluated (Table 2). The most effective anticancer agent, 5-fluorouracil (5-FU) with 13.35 µg/mL concentration was used as a reference drug. The results showed that heterocyclic curcumin-based derivative (compound **8**) exhibited remarkable anticancer activity against MCF-7 cell line which displayed in vitro cytotoxic activity with an IC_50_ value of 20 µg/mL for the MCF-7 cell line, whereas compound **7**, **9**, and **10** with IC_50_ values of 33 µg/mL, 33 µg/mL and 37 µg/mL, respectively, showed no effect on the MCF-7 cell line. Therefore, the results compared to the reference anticancer drug demonstrated that compound **8** displayed a moderate to high growth-inhibitory action on the tested cell line ranged from 0 to 50 µg/mL concentrations [1].

**Scheme 3:**
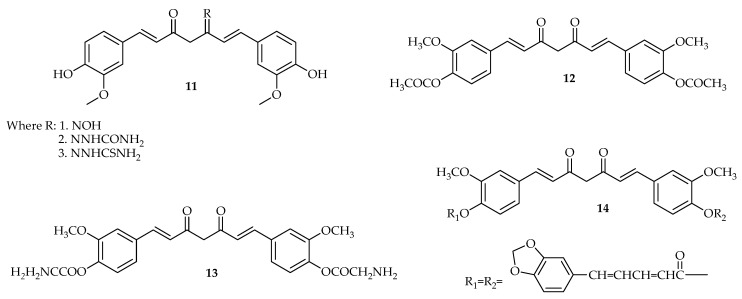

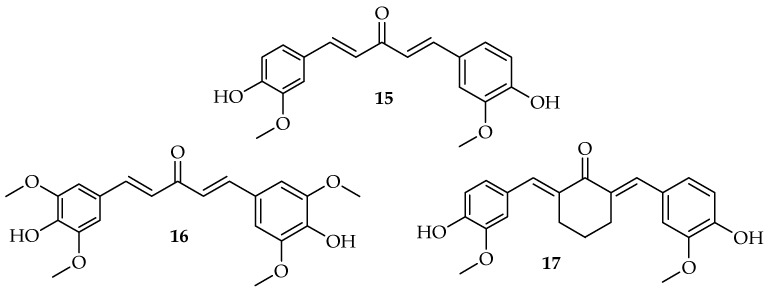
Compounds 11–17.

Agrawal and Mishra synthesized new curcumin derivatives, in these compounds; derivative (**11**) exhibited significant antioxidant activity. These curcumin derivatives were also evaluated for antiproliferative effects against MCF-7 estrogenic-dependent breast cancer cell line when compared to curcumin alone with 64% cell viability. The result revealed curcumin derivative as an effective antiproliferative agent with 26% cell viability [57]. Compound **13** and **14** displayed increased activity which indicates that these curcumin derivatives may be effective and may have the ability to overcome the bioavailability problem that is faced by free curcumin (Scheme 3). The curcumin derivatives **15** and **16** displayedsignificant anticancer action when compared to curcumin alone in various ER+ and ER− human breast cancer cells. The IC_50_ values of **15** and **16** ranged from 0.3 to 5.7 µM, respectively, which is lower than the IC_50_ values of curcumin (1–7.5 µM) cell lines. Compound **15** and **16** inhibited Akt, STAT3, and HER2/ Neu pathways and induced apoptosis at the concentration of 10 µM [58]. These compounds were also active when combined with doxorubicin as they showed a synergistic antiproliferative effect in MDA-MB-231 with the IC_50_ value of 2.8 and 2.7 µM, respectively, on breast cancer cell lines. Moreover, these compounds inhibited anchorage-independent advance and cell migration in MDA-MB-231 cells (Table 3) [58]. Compound 1**5** was found to be cytotoxic toward ER-breast cancer cells with the IC_50_ value of 5.0 μM and exhibited antiangiogenic effects in human and murine endothelial cell lines [58,59].

**Scheme 4:**
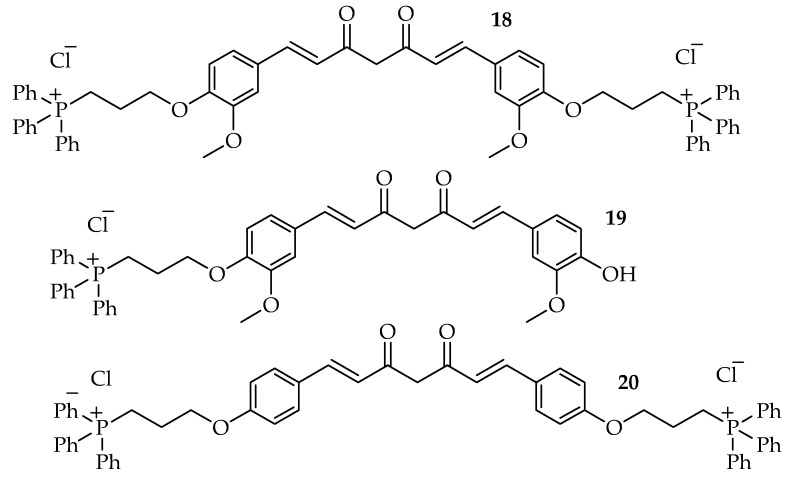
Compounds 18–20.

The cytotoxic effects of the derivatives were observed and compared with the free curcumin. Compound **18** IC_50_ of 2.31 μM, in particular, showed the most potent activity when compared to that of curcumin with an IC_50_ value of 40.32 μM against MCF-7 cell line followed by **20** with an IC_50_ value of 3.84 μM. The IC_50_ value of compound **19** was 5.31 μM (Table 4). Since compound **18** and **20** were found to be more potent, it may be due to the absence of OH group in compounds which indicate that the OH group does not affect the uptake and cytotoxic effect of the derivative towards cancer cell (Scheme 4) [60]. The antiproliferative potential of curcumin derivatives **21**–**24** was determined using MCF-7 cell lines (Table 5). The results of half-maximal proliferation inhibitory concentration of **21**, **22**, **23**, and **24** were found to be 1.5 ± 0.7, 26.2 ± 3, 2.9 ± 0.4, and 6.3 ± 0.2 μM, respectively, on MCF-7 cell lines. The curcumin inhibited the proliferation of MCF-7 cells with an IC_50_ value of 17.1 ± 0.7 μM [61]. These results indicate that **21** and **24** were more effective inhibitors of MCF-7 proliferation when compared to curcumin alone. Compound **21** can target many cancer cells at a low concentration indicating that this compound has a strong anticancer activity [61].


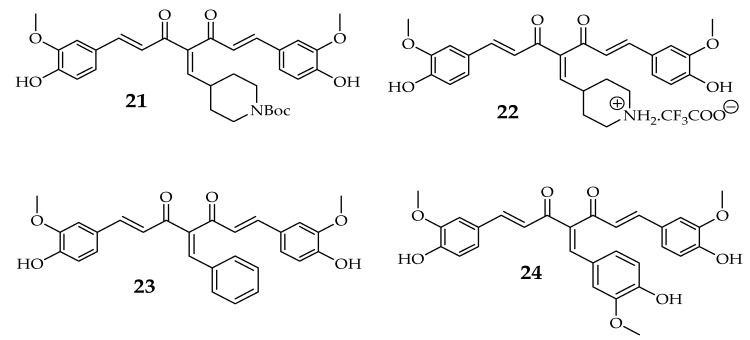


#### 3.1.2. Clinical Studies of Curcumin Derivatives in Breast Cancer

Bayet-Robert et al. established the tolerability, possibility, and safety of the combination of curcumin with docetaxel in metastatic and advanced breast cancer patients [62]. A total of 14 patients were treated with docetaxel chemotherapy, (1 h-perfusion, 100 mg/m^2^ every 3 weeks for six cycles) for a total of 63 cycles of treatment. Docetaxel was decreased to four patients at the dose of 75 mg/m^2^ from day one of treatment to avoid toxicity events in elder patients. A total of six dose levels (DLs) of curcumin were tested. The evaluation of curcumin, in combination with docetaxel, was the first clinical trial in metastatic and advanced breast cancer patients. For Phase I trial, the maximally tolerated dose (MTD) of curcumin was 8000 mg/day, and for docetaxel in the combination, it was 100 mg/m^2^ every 3 weeks for six cycles [56]. Curcumin was given orally for seven successive days for six cycles. In the earlier studies of phase I, curcumin was found to be safe at a dose of 8000 mg/day. For breast cancer patients, docetaxel was used as a monotherapy in metastatic breast cancer at a conventional dose in anthracycline pretreated. The tested combination of hematological toxicity showed no increased incidence. Vascular endothelial growth factor (VEGF) overexpression is clinically associated with larger tumor size, increased metastasis, and poor prognosis in metastatic breast cancer patients. The combination of curcumin and docetaxel significantly reduced VEGF levels after three cycles of treatment. The investigation of the phase of the randomized clinical trial confirmed the effectiveness of the combination of curcumin with other anticancer drugs in metastatic [62].

### 3.2. Prostate Cancer

American Cancer Society (ACS) stated that prostate cancer is the second cause of cancer-related deaths in American men [63]. In 2013, new cases of prostate cancer were estimated to about 235,000, [64]. Recently, the American Cancer Society reported an estimate of 174,650 cases of prostate cancer. The aforementioned statistics makes it the second most common cause of cancer death in males [65]. The present treatment procedures for prostate cancer that have been used include radiation therapy or combination therapy, non-steroidal antiandrogens, and administration of steroidal, chemotherapy, and surgery. Even though these different option treatments can be effective in controlling the development of prostate cancer, they are also associated with other diseases that affect sexual and urinary function. Hence, prostate cancer research is aimed at developing advanced treatment options to avoid some complications [64]. Androgen ablation therapy is one of the therapeutic agents for prostate cancer which prevent androgen receptor (AR) function. Combining AR inhibition and androgen synthesis suppression can be used as an effective, aggressive form of therapy [65]. Hence, the identification of mechanisms and chemical agents that prevent AR signaling warrant thorough investigation for the improvement of new prostate cancer drugs [66]. Several studies demonstrated curcumin as an effective agent to induce apoptosis and hinder proliferation in prostate cancer for both in vitro and in vivo studies by interfering with several cellular pathways, such as nuclear factor κ (NFκB), epidermal growth factor (EGFR), and mitogen-activated protein kinase (MAPK) [20]. Its low bioavailability, low cancer-killing potency, and multiple biological effects have resulted in the design of curcumin derivatives with enhanced solubility and anticancer activity [20]. Since curcumin has a low bioavailability, the concentrations (IC_50_, 20 µM) needed to exert its anticancer activity are not easy to reach in the blood plasma of the patients. Hence, significant effort has been made in the development of derivatives of curcumin which with potent anticancer properties characterized by a lower IC_50_ value when compared to curcumin [63]. Yang et al. synthesized EF24 curcumin derivative with enhanced antitumor activity when compared to curcumin, but the therapeutic efficacy and mode of action are still not known which is significant to address as curcumin targets several signaling pathways [67]. Another curcumin derivative which has been used for prostate cancer treatment is dimethylcurcumin which improves AR degradation [68]. The structure-activity relationship (SAR) study for derivatives of curcumin shows that the existence of a β-diketone and a coplanar hydrogen donor group hybrid is important for the antiandrogenic activity for the remedy of prostate cancer [69].

#### 3.2.1. Curcumin Derivatives as Prostate Cancer Inhibitors

**Scheme 5:**
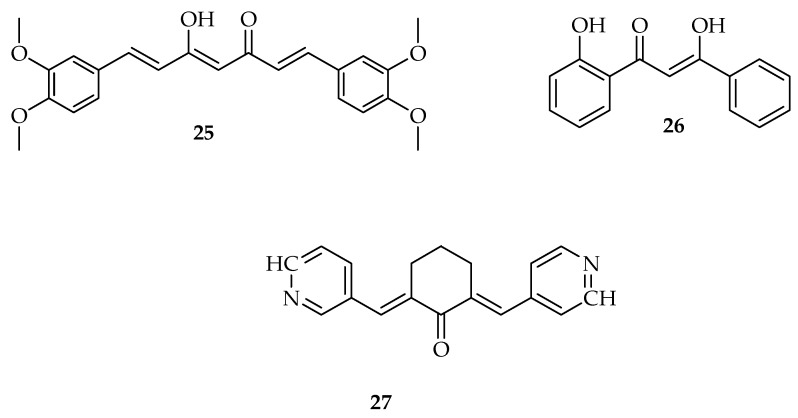
Curcumin derivatives with anti-breast cancer activity 25–27.

**Figure 4:**
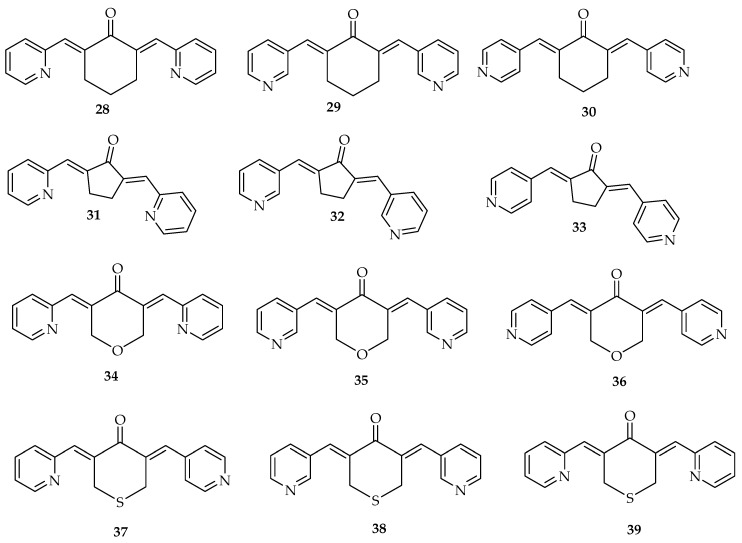
Pyridine derivatives of curcumin as **28**–**30** and **37**–**39**.

**Figure 5:**
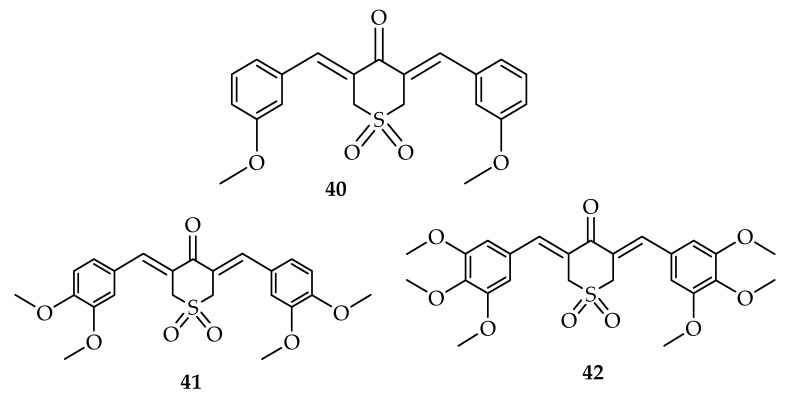
Curcumin derivatives containing sulfone (**40**–**42**).

**Figure 6:**
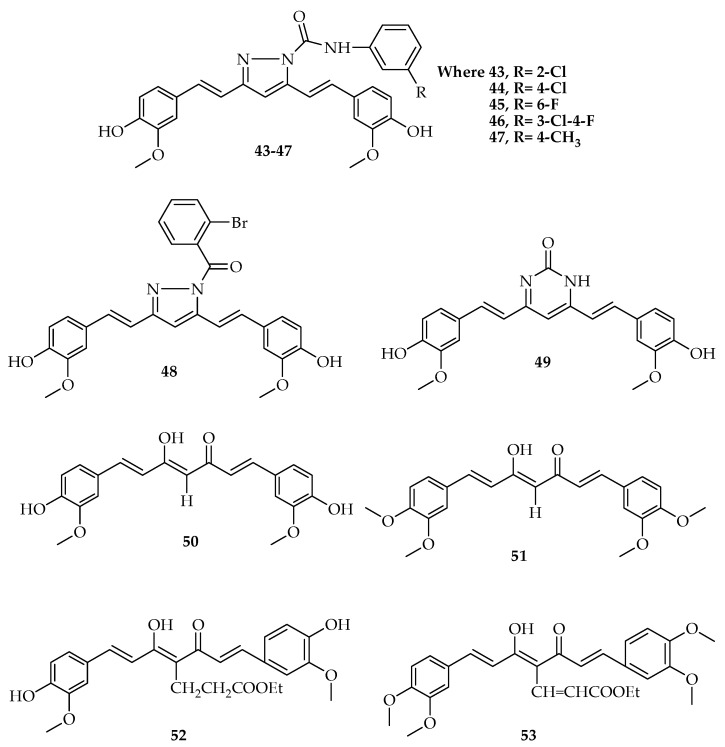
Curcumin derivatives, **43**–**53** effective against prostate cancer.

Pyridine derivatives of curcumin were prepared and tested against CWR-22Rv1 prostate cancer cell line (Scheme 5). All tested derivatives exhibited high inhibitory effect better than curcumin (IC_50_ = 16.99 µM). In the sets of four pyridine derivatives of curcumin, **28**–**30** and **37**–**39** (Table 6) demonstrated the highest potent inhibitory efficacy against the CWR-22Rv1 growth of cultured cells [63]. The IC_50_ values for the **25**–**27** (Scheme 5) and **34**–**36** groups (Figure 4) were smaller than 1 µM against CWR-22Rv1 cells, which indicate that these derivatives were approximately 20 fold more effective than that of curcumin (IC_50_ = 16.99 µM). The IC_50_ values of these sets of four pyridine derivatives of curcumin ranged from 0.49 to 4.99 µM, respectively [63]. Curcumin derivatives containing sulfone have been investigated (**40**–**42**) against numerous cancer cell lines including prostate cancer PC-3 cells, lung cancer H1299 cells, colon cancer HT-29 cells, and pancreatic cancer BxPC-3 cells (Figure 5) using 3-(4,5-dimethylthiazol-2-yl)-2,5-diphenyltetrazolium bromide (MTT assay) [30]. The values of IC_50_ for the compounds ranged from 0.72 μM to 1.73 μM on PC-3 cells, whereas the IC_50_ value of curcumin was 21.54 μM, 0.46 μM to 1.24 μM on H1299 cells, 0.19 μM to 0.38 μM on HT-29 cells, and 0.29 μM to 1.01 μM on BxPC3 cells. According to the results, curcumin derivatives (**40**–**42**) were more effective compared to curcumin alone (Table 7). Compound **42** with an IC_50_ value of 0.72 μM followed by compound **41** with an IC_50_ value of 0.85 μM on PC-3 cells exhibits promising results for further in vivo studies for anticancer activities in suitable animal models [30]. The derivatives (**43**–**49**) were found to be effective, and three-dose response parameters (GI_50_, TGI, and LC_50_) were calculated for each of the experimental agents (Figure 6). The compound **40** exhibited the highest sensitivity to PC-3 cells with GI_50_ of 0.31 µM. The best value of TGI was being noted on compound **44** with 1.47 µM. For all compounds except compounds **40**, **44,** and **47**, the LC_50_ value was > 100 µM [70].

Elias et al. synthesized curcumin derivatives (**50**–**53**) which demonstrated effectiveness in vitro cytotoxic activity against PC-3 and LNCaP human prostate cancer cell lines (Figure 6) [71]. Compound **53** displayed the most effective activity on LNCaP cell line with an IC_50_ value of 0.2 µM, the same compound was found to be effective against PC-3 cell lines with an IC_50_ value of 1.0 µM. Compound **51** and **52** were active against LNCaP cell line with an IC_50_ value of 1.3 and 1.5 µM, respectively. Compound **51** showed potent activity against PC-3 cell lines with an IC_50_ value of 1.1 µM. The results suggest that compound **51**–**53** exhibit anti-prostate cancer activity (Table 8) [71].

**Figure 7:**
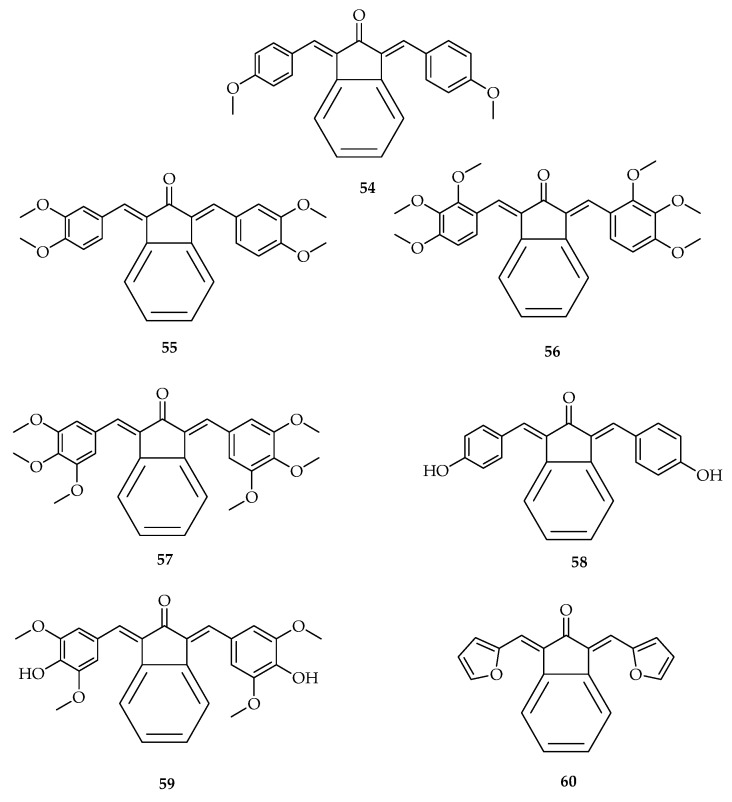
Curcumin derivatives (**54**–**60**) effective against cultured prostate cancer PC-3 cells and nontumorigenic human prostate epithelial RWPE-1 cells.

The inhibitory effect of seven curcumin derivatives (**54**–**60**) on the growth of cultured prostate cancer PC-3 cells and nontumorigenic human prostate epithelial RWPE-1 cells were determined, and curcumin was assessed as a positive control in each incubation (Figure 7). Most of the compounds showed potent inhibitory effects when compared to curcumin [72]. Compound **57** was found to exhibit effective inhibitory effects against PC-3 cell with an IC_50_ value of 0.64 ± 0.1 µM when compared to curcumin with an IC_50_ value of 19.98 ± 2.4 µM. The IC_50_ values of compound **59**, **55**, **60**, **54**, **58,** and **56** were 2.46 ± 0.3, 3.05 ± 0.4, 8.12 ± 0.9, 8.30 ± 0.9, 9.6 ± 1.1, and 10.06 ± 1.3 µM, respectively, in PC-3 cell lines (Table 9) [72]. The IC_50_ value of compound **59** and **57** were 4.2 ± 0.5 and 9.12 ± 0.4 µM, respectively, while that of curcumin was 15.62 ± 1.5 µM, which exhibited an inhibitory effect against RWPE-1 cells. The other compounds did not show any inhibitory effect in RWPE-1 cell line, the IC_50_ values ranged from 18.13 ± 5.4 to 39.26 ± 5.1 µM. The compounds exhibited lower cytotoxicity (higher IC_50_) in RWPE-1 cells when compared to PC-3 cells. The IC_50_ value of compound **57** in RWPE-1 cells was higher than in PC-3 cells which indicates that compound **57** is more toxic to cancer cells than non-cancer cells [72].

#### 3.2.2. Clinical Studies of Curcumin Derivatives on Prostate Cancer

Hejazi et al. conducted a randomized, double-blinded, placebo-controlled clinical trial on the effect of curcumin on the oxidative status of patients with prostate cancer during radiotherapy. Forty patients with prostate cancer were administered a capsule containing 347 mg of curcumin, 84 mg of desmethoxycurcumin, 9 mg of bisdemethoxycurcumin, and each placebo capsule of 500 mg. Patients received 2.6 g of curcuminoids per day, 2 g of curcumin per day, and placebo of two capsules with each meal for one week before the start and during the radiotherapy [73]. Twenty patients from each curcuminoids group and placebo group finished the study, and they included the final study for both groups. Plasma total antioxidant capacity (TAC), catalase activity, glutathione peroxidase activity (GPx), and superoxide dismutase (SOD) were measured for oxidative status for one week before initiation of radiotherapy and three months after radiotherapy. After radiotherapy, a significant TAC increase was observed while the activity of SOD decreased compared with those at baseline, which indicates an antioxidant effect of curcumin [73]. The catalase activity and GPx did not reveal any significant changes. Treatment effects were assessed by serum prostate-specific antigen (PSA) levels. In both groups, the treatment was effective. In both groups, the PSA levels at baseline were 12.98 ± 7.09 ng/mL and 16.47 ± 5.94 ng/mL, respectively. The PSA levels were decreased to 0.12 ± 0.16 ng/mL and 0.13 ± 0.06 ng/mL, respectively, compared to the baseline levels in both groups after completion of radiotherapy. Their results revealed that curcumin could increase TAC and reduce SOD activity in the plasma of patients with prostate cancer and the patients that are receiving radiotherapy. Regarding the treatment outcomes, no significant differences were noted between the two groups [73].

### 3.3. Colon Cancer

In the United State, colon cancer is the second most common cause of deaths. According to their statistics, in 2006, about 55,000 deaths were caused by colon cancer [74]. Colon cancer is among the most chronic cancer in humans. It is the third most commonly treated cancer in males, and the second treated cancer in females worldwide [75,76]. In developing countries, the lack of sufficient access and limited treatment standard contribute to the increasing rate of death caused by colon cancer [75]. The occurrence of colon cancer is also associated with a genetic predisposition. People with relatives who have had colon cancer are at a higher risk of developing the disease than folks with no family history. If one or more relatives are diagnosed with colon cancer at a very young age, the risk chances are three to six times more than that of the general population [77]. Around 20% of all colon cancer patients have close relatives who have been diagnosed with the disease [78]. Around 5% of colon cancer patients have a well-defined genetic-syndrome that causes the disease [79]. Patients with other chronic diseases, such as irritable bowel syndrome (IBS), ulcerative colitis, and Crohn’s disease, are at a higher risk of developing colon cancer [80]. There are also other risk factors, such as type 2 diabetes, obesity, physical inactivity, drinking alcohol, and smoking. Consumption of a diet high in processed meat can increase the chances of having colon cancer. Diets low in fruits, vegetables, and fiber are linked with a higher risk of developing colon cancer [81,82] Chemotherapy is one of the most practiced treatment approach employed in metastatic condition [83]. However, patients identified with colon cancer undertake surgical elimination of the cancer tissue with chemotherapy, and over half of those patients suffer from relapse [84,85]. Moreover, the clinical application of these chemotherapeutic agents suffers from serious side-effects, such as toxicity and resistance development by the cancer cells [75]. Since therapies, such as radiation, chemotherapy, and surgical resection, are often insufficient for the treatment of disease, the development of new treatment options has increased [74]. Researchers have found that many natural products that are purified and their derivatives exhibit distinct biological and pharmacological activities, making them the potent drug for tumor treatment [75]. Curcumin was tested, and the clinical studies revealed that curcumin has anticancer and antiangiogenic activities. Its anticancer activities include apoptosis induction and cancer growth inhibition in a variety of cultured tumor tissue in vitro. Moreover, curcumin has displayed the capability to prevent tumorigenesis in vivo [74]. Additionally, curcumin has effects on numerous different goals, including adhesion molecules, transcription factors, growth regulators, cellular signaling molecules, and angiogenesis regulators [75]. Curcumin displays promising in vitro results in chemotherapeutic and chemo-preventive effects in all different types of cancers.

#### 3.3.1. Curcumin Derivatives as Colon Cancer Inhibitors

**Figure 8:**
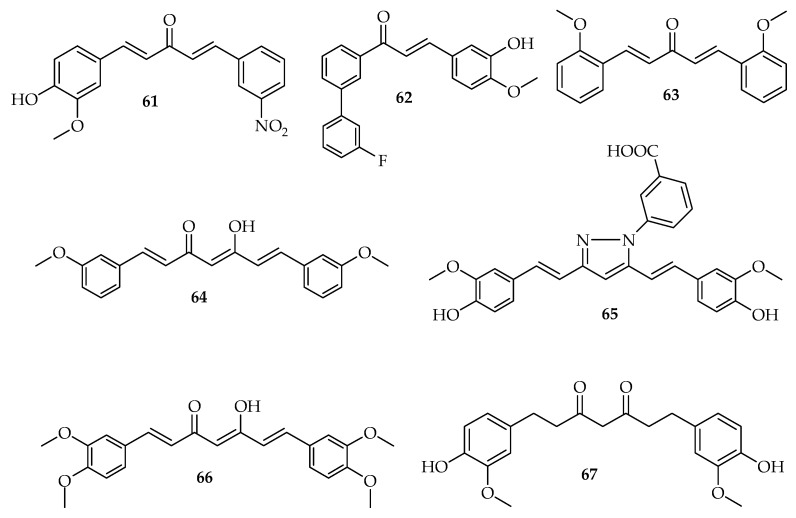
Curcumin derivatives with anti-colon cancer activity **62**–**67**.

In vitro assay results showed that curcumin derivatives exhibited enhanced antiproliferative effects against colon cancer cell lines when compared to curcumin alone. Zheng and colleagues synthesized the mono carbonyl derivative of curcumin (WZ35) (**61**) (Figure 8) to increase the therapeutic efficacy and the bio-availability of curcumin. The in vitro studies showed that WZ35 displayed greater antiproliferative effects on colon-cancer cells when compared to curcumin [86]. The in vitro assays of another curcumin derivative, bis-DeHydroxy curcumin (bDHC) (**64**) has been reported to induce the autophagy on colon-cancer cells [37]. Dimethoxy-curcumin (DMC) (**66**), a lipophilic derivative of curcumin, with methylated phenolic hydroxyl groups has greater chemical and metabolic stability when compared to curcumin [19,87]. Additionally, DMC is a potential anticancer agent which induces the apoptosis in colon cancer cells with less toxicity to normal cells and has better bioactivity when compared to curcumin [88,89].

**Scheme 6:**
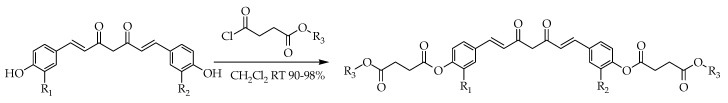
Synthesis of succinate derivatives of curcuminoids (**69**–**73**).

Other studies demonstrated that curcumin derivative, tetrahydrocurcumin (THC) (**67**) is more effective when compared to curcumin in terms of the inhibition of the aberrant crypt foci (ACF) development and cell proliferation [90]. Monocarbonyl curcumin derivative MC37 (**61**) did not only inhibit the growth of colon cancer cells but also blocked the cell-cycle progression at G2/M phase [91]. Conjugation of succinic acid derivatives with curcuminoids has also been reported in drug discovery of anti-colon cancer agents. Wichitnithad et al. synthesized a series of six succinyl derivatives of three curcuminoids (curcumin, bisdesmethoxycurcumin and desmethoxycurcumin) via aldol-condensation of pentane-2,4-dione with different benzaldehydes. The curcuminoid derivatives **69**–**73** (Scheme 6, Table 10) carrying succinyl ester moieties showed enhanced stability and anti-colon cancer activity. The synthesized derivatives had IC_50_ values ranging from 1.8 μM to 9.6 μM, compared to IC_50_ values of the parent compounds ranging from 3.3 μM to 4.9 μM. Curcumin diethyl disuccinate (**68**) exhibited the highest potency [92].

Curcumin and its derivatives were found to be effective in terms of targeting chemo-resistant colon cancer cells. Modified derivatives of curcumin were also synthesized with intentions of achieving better stability. Curcumin derivatives have been investigated in various cancers and have been proven to be safe [51].

#### 3.3.2. Clinical Studies of Curcumin Derivatives

The data reporting the pharmacokinetic properties of curcumin derivatives in humans is very limited. However, the study of pharmacokinetics, toxicology, and effective biological dose of curcumin have been reported. Some researchers have reported the molecular targets of curcumin derivatives (Table 11). Cheng and co-workers conducted a clinical trial on 25 patients with precancerous-lesions and the free curcumin concentrations (mean ± SD) in plasma after taking 4000, 6000, and 8000 mg of curcumin daily for 3 months were 0.51 ± 0.11 µM, 0.63 ± 0.06 µM, and 1.77 ± 1.87 µM, respectively [93]. In another study of six patients with advanced colon cancer treated with 3.6 g of curcumin per day for three months yielded 4.3 µg/L, 5.8 µg/L, and 3.3 µg/L mean plasma-concentrations of curcumin and its derivatives curcumin-glucuronide and curcumin-sulfate respectively, 1 h after administration [94].

## 4. Conclusions and Future Perspectives

Curcumin, a naturally occurring therapeutic agent, possesses notable biological activities, such as antioxidant, antimicrobial, anticancer, and anti-inflammatory activity. In terms of cancer treatment, curcumin is a modulator for multiple targets at different stages of cancer progression, such as proliferation, metastasis, angiogenesis, and apoptosis. However, its poor bioavailability and poor pharmacokinetic profile in a clinical application result in its low anticancer potency.

In this context, curcumin-related derivatives pose a new-platform for chemotherapy, and it is evident that curcumin derivatives overcome the aforementioned limitations and improve therapeutic efficacy. The underlying mechanism of curcumin derivatives as anticancer agents also follows proliferation inhibition and apoptosis induction in various cancer cell lines. Although several curcumin derivatives with enhanced anticancer activity have already been reported, new curcumin derivatives still need to be synthesized or developed to further enhance its anticancer activity. Additionally, there is a pressing need for a thorough research to understand the mode of action of these derivatives. The anticancer effects of curcumin and its derivatives can be synergistically improved by applying them in combination with other new anticancer drugs. Another useful synthetic strategy could be the synthesis of conjugates by coupling curcumin and its derivatives with other chemotherapeutic agents, such as pullulan [99], cisplatin [100], etc.

## Figures and Tables

**Table 1:** The in vivo and in vitro studies showing some molecular targets of curcumin.

Types of Cancer	Molecular Targets of Curcumin	References
Breast cancer	Regulates the apoptosis, cell phase-related genes, and micro-RNA in breast cancer cells.	[38]
	Modulates carcinogenesis of the breast.	[39]
	Inhibits the proliferation of BCSC (breast cancer stem cells).	[40]
	Inhibit the stem-like properties and regulates the EMT (epithelial-mesenchymal transition) process.	[41]
Prostate cancer	Inhibits proliferation and the ability of colony formation of prostate cancer cells.	[42]
	Inhibits phosphorylation of downstream targets of the LNCaP cells.	[43]
	Inhibits the NF-κb-regulate gene products in the DU-145 cells.	[44]
	Inhibits the expression of the androgen receptor of the LNCaP cells.	[45]
Colon cancer	Suppresses the oncogenicity of human-colon cancer cells in human colon-cancer cells.	[46]
	Inhibits AP-1 and NF-κB signaling pathways, suppress JNK activation induced by carcinogens.	[47]
	Inhibits PKC activation by inhibiting the release of Ca^2+^ from the endoplasmic reticulum.	[48]
	Inhibits carcinogenesis in various types of cancer including colorectal cancer and curcumin is able to inhibit the inflammatory response and the oxidative stress and to induce apoptosis in cancer cells	[49]
	Suppresses the expression of EGFR, mediated by the reduction of Egr-1 activity in Caco2, and HT29 colon cancer cells, inhibiting colon cancer cell growth.	[50]
	Reduce the size of tumor mass and growth in both in vivo and in vitro studies by affecting many intracellular events that are associated with cancer progression and cancer stem cells formation.	[51]
	Suppresses LPS-induced cyclooxygenase-2 gene expression by inhibiting AP-1 DNA binding in BV2 microglial cells.	[52]
	Inhibits the cell proliferation followed by suppression of EGFR gene and cyclin D1 gene expression.	[53]

**Table 2:** The inhibitory concentration of curcumin derivatives **7**–**10**.

Compounds	IC_50_ µM
	MCF-7
**7**	33
**8**	20
**9**	33
**10**	37

**Table 3:** The inhibitory concentration of curcumin and its derivatives **15**–**17**.

Compounds	IC_50_ µM				
	MCF-7	MDA-MB-231	MDA-MB-468	MDA-MB-453	SKBr3
**1**	-	7.6	1	-	-
**15**	2.4	2.8	0.3	4.7	5.7
**16**	1.7	2.7	0.3	1.3	3.8
**17**	-	5	-	-	-

**Table 4:** The inhibitory concentration of curcumin and its derivatives **18**–**19**.

Compounds	IC_50_ µM
	MCF-7
**1**	40.32
**18**	2.31
**19**	5.31
**20**	3.84

**Table 5:** The inhibitory concentration of curcumin and its derivatives **21**–**24**.

Compound	IC_50_ µM
	MCF-7
**1**	17.1 ± 0.7
**21**	1.5 ± 0.7
**22**	26.2 ± 3
**23**	2.9 ± 0.4
24	6.3 ± 0.2

**Table 6:** The inhibitory concentration of curcumin and its derivatives **28**–**39**.

Compounds	IC_50_ µM
	PC-3
**1**	16.99 ± 2.1
**28**	0.53.± 0.1
**29**	0.92 ± 0.1
**30**	0.95 ± 0.2
**31**	4.75 ± 0.5
**32**	4.99 ± 0.5
**33**	3.03 ± 0.4
**34**	2.18 ± 0.2
**35**	1.07 ± 0.1
**36**	1.80 ± 0.2
**37**	0.66 ± 0.1
**38**	0.55 ± 0.1
39	0.49 ± 0.1

**Table 7:** The inhibitory concentration of curcumin and its derivatives **40**–**42**.

Compounds	IC_50_ µM			
	PC-3	H1299	HT-29	BxPC-3
**1**	21.64 ± 1.83	19.87 ± 0.94	18.39 ± 0.35	18.25 ± 1.27
**40**	1.73 ± 0.26	1.24 ± 0.08	0.19 ± 0.14	1.01 ± 0.11
**41**	0.85 ± 0.10	0.58 ± 0.04	0.38 ± 0.15	0.32 ± 0.08
**42**	0.72 ± 0.17	0.46 ± 0.01	0.29 ± 0.09	0.29 ± 0.09

**Table 8:** The inhibitory concentration of curcumin and its derivatives **50**–**53**.

Compounds	IC_50_ µM	
	PC-3	LNCaP
**50**	7.7	3.8
**51**	1.1	1.3
**52**	5.1	1.5
**53**	1.0	0.2

**Table 9:** The inhibitory concentration of curcumin and its derivatives.

Compounds	IC_50_ µM	
	PC-3	RWPE-1
**1**	19.98 ± 2.4	15.62 ± 1.5
**54**	8.30 ± 0.9	39.26 ± 5.1
**55**	3.05 ± 0.4	18.13 ± 5.4
**56**	10.06 ± 1.3	18.44 ± 1.1
**57**	0.64 ± 0.1	9.12 ± 0.4
**58**	9.6 ± 1.1	29.23 ± 3.9
**59**	2.46 ± 0.3	4.2 ± 0.5
**60**	8.12 ± 0.9	27.66 ± 2.3

**Table 10:** The **IC_50_** values of succinate derivatives of curcuminoids.

Compounds	R1	R_2_	R_3_	IC_50_ (μM) ± SD)
**68**	OMe	OMe	Me	3.84 ± 0.19
**69**	OMe	OMe	Et	1.84 ± 0.11
**70**	H	H	Me	3.78 ± 0.31
**71**	H	H	Et	5.97 ± 0.28
**72**	OMe	H	Me	4.40 ± 0.15
**73**	OMe	H	Et	9.60 ± 0.31

**Table 11:** The in vivo and in vitro studies showing molecular targets of curcumin derivatives.

Type of Cancers	Curcumin Derivatives	Molecular Targets	References
Breast cancer	**28**	Inhibits many different types of steroid receptors in breast cancer cells	[26]
	**29**	On MCF-7, reduce the number of cells and induced shrinkage of cells. It significantly downregulated the expression of PLK1, whereas improved the appearance of p21 and WEE-1	[95]
	**30**	Induces G_2_/M-phase cell cycle arrest and apoptosis significantly. It modulates the expression of main cell signaling proteins, precisely, in AKt, SKBr3 cells, and protein levels of Her-2.	[17]
	**15** and **16**	Inhibits AKt, STAT3, and HER2/Neu pathways and also	[58]
		induced apoptosis at IC_50_ value of 10 µM.	
	**2**–**6**	Prevents the development of breast cancer stem cell growth by decreasing P-gp mediated efflux process	[8]
	**18**	Inhibits Akt and STAT3 phosphorylation and significantly increased ERK phosphorylation.	[60]
	**21**	Induces p53 mediated apoptosis against MCF-7 cells. In MCF-7 cells, it disturbs microtubules and induces p53 dependent apoptotic cell death	[61]
Prostate cancer	**28**	Increases androgen receptor degradation in androgen-dependent prostate cancer cells.	[20]
		Reduces ARs with the F876L mutation in DU-145 and C4-2 cells, and destroys prostate cancer stem/progenitor (S/P) cell invasion through the alteration of EZH2/STAT3 signaling in mice with CWR-22Rv1 CD133+ S/P xenografts.	[96]
	**40**–**42**	Reduces ARs with the F876L mutation in DU-145 and C4–2 cells, and destroy prostate cancer stem/progenitor (S/P) cell invasion through the alteration of EZH2/STAT3 signaling in mice with CWR-22Rv1 CD133+ S/P xenografts.	[96]
		Reduces the level of phosphorylated signal transducer and activator of transcription-3 (p-STAT3) (Tyr705).	[30]
	**68** and **69**	Prevents the growth of androgen-dependent and -independent prostate cancer cells with a sub micromolar range of IC_50_ values.	[97]
Colon cancer	**61**	Inhibits cell the proliferation of colon cells.	[13]
	**63**	Shows antiproliferative effects. Induces the cell cycle arrest, the necrosis, and the apoptosis in human colon cancer.	[86]
	**64**	Induces the autophagy and enhances the antiproliferative activity on colon cancer cells.	[98]
	**66**	Inhibits the proliferation and inducing apoptosis.	[25,87,88,89]
	**62**	Inhibits the growth of colon cancer cells and blocks cell cycle progression.	[91]
	**67**	Inhibits the aberrant crypt foci (ACF) development and cell proliferation.	[90]
